# Ultrasound before instrumental vaginal delivery: A useful tool to avoid misdiagnosis of fetal head position

**DOI:** 10.1111/aogs.14463

**Published:** 2022-09-21

**Authors:** Ilenia Mappa, Francesco D'Antonio, Tullio Ghi, Giuseppe Rizzo

**Affiliations:** ^1^ Department of Obstetics and Gynecology, Fondazione Policlinico Tor Vergata Università di Roma Tor Vergata Rome Italy; ^2^ Department of Obstetrics and Gynecology Università di Chieti Chieti Italy; ^3^ Department of Obstetrics and Gynecology Università di Parma Parma Italy

*Sir*,

We thank Dr. Liu et al.^1^ for their letter on our systematic review^2^ and for the opportunity to discuss this debatable topic even further. Our study has investigated whether the use of intrapartum ultrasound before instrumental vaginal delivery might increase the success rate of the procedure and decrease the occurrence of adverse effects. The data from the available randomized trials were pooled in a meta‐analysis, showing no significant decrease in the variables considered, except in the improved rate of correct diagnosis of fetal head position. We totally agree with Dr. Liu and colleagues that our systematic review does not fully elucidate whether ultrasound may be beneficial or detrimental before instrumental vaginal delivery. Limitations related to small sample size, an even smaller number of events and dissimilarity in the included populations are likely to bias our results, as we stressed in our study. However, the main aim of a systematic review is not to provide definitive evidence but rather to show whether an objective finding is present on a given topic.

In our systematic review, the risk of having an incorrect diagnosis of fetal head position was lower when ultrasound was performed before instrumental delivery, with a relative risk of 0.16 (95% confidence interval [CI] 0.1–0.3; *p* < 0.001). The rate of failed vaginal operative delivery is reported in the literature as around 5% and is consistently associated with suboptimal maternal and perinatal outcomes. More importantly, incorrect diagnosis of fetal head position is among the main determinants of failed instrumental vaginal delivery.^3^ Unfortunately, we could not assess whether the role of intrapartum ultrasound might improve the clinical outcomes when an incorrect diagnosis of fetal position had been clinically made because the original studies did not report such a comparison. Trials exploring the potential role of ultrasound in labor are commonly underpowered to detect differences in maternal and perinatal outcomes, and this translates into a lack of statistical but not clinical significance.

The reported higher diagnostic accuracy of ultrasound compared with standard examination in correctly identifying the fetal head position is crucial because it may potentially lead to a better execution of operative delivery, reducing the number of tractions, failed applications and, in turn, improving maternal and perinatal outcomes. This also explains why recent guidelines strongly support the use of ultrasound before an operative vaginal delivery despite the lack of large trials demonstrating its actual beneficial role.^4^

Ultrasound is better tolerated than clinical examination and this compliance increases in the case of an unplanned operative delivery.^5^ On this basis, it is the collective authors’ opinion that the use of ultrasound in labor should be encouraged before a trial of operative delivery. Large and adequately powered trials looking at the role of ultrasound assessment before operative delivery in those cases in which fetal head position and station have not been correctly identified at clinical examination, are needed to support this statement and elucidate whether this may translate into a better intrapartum care.
